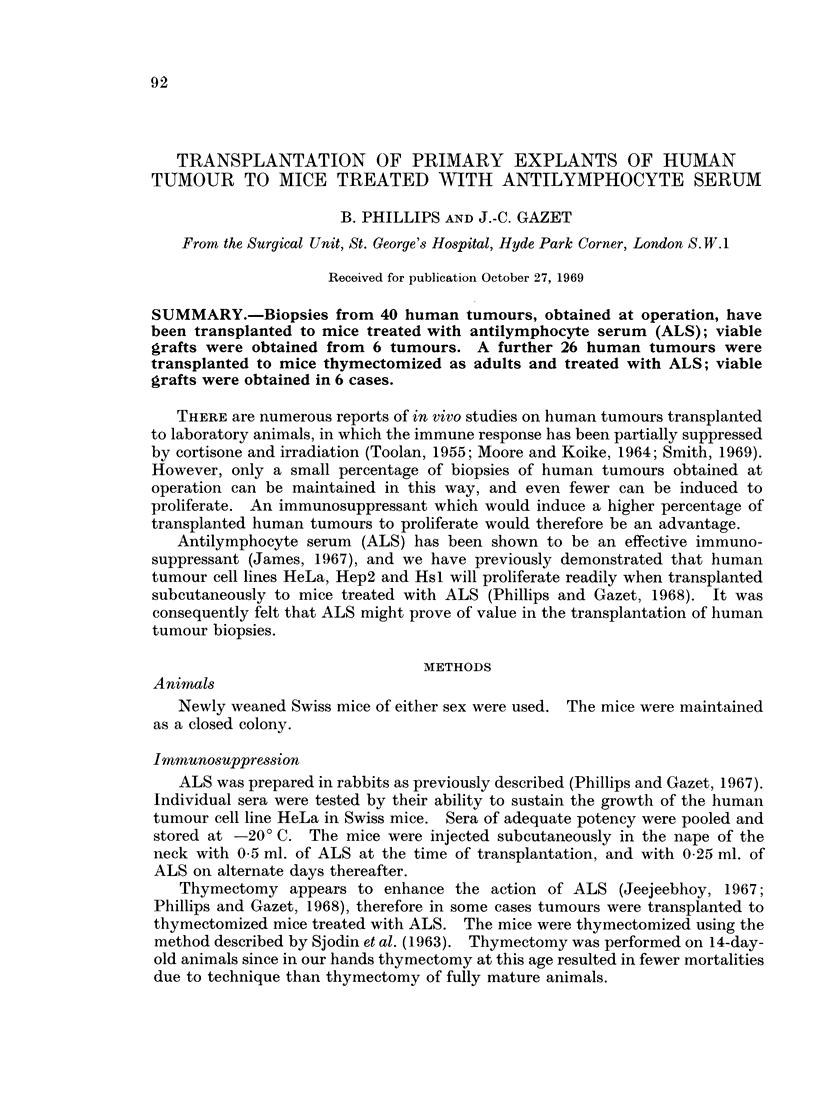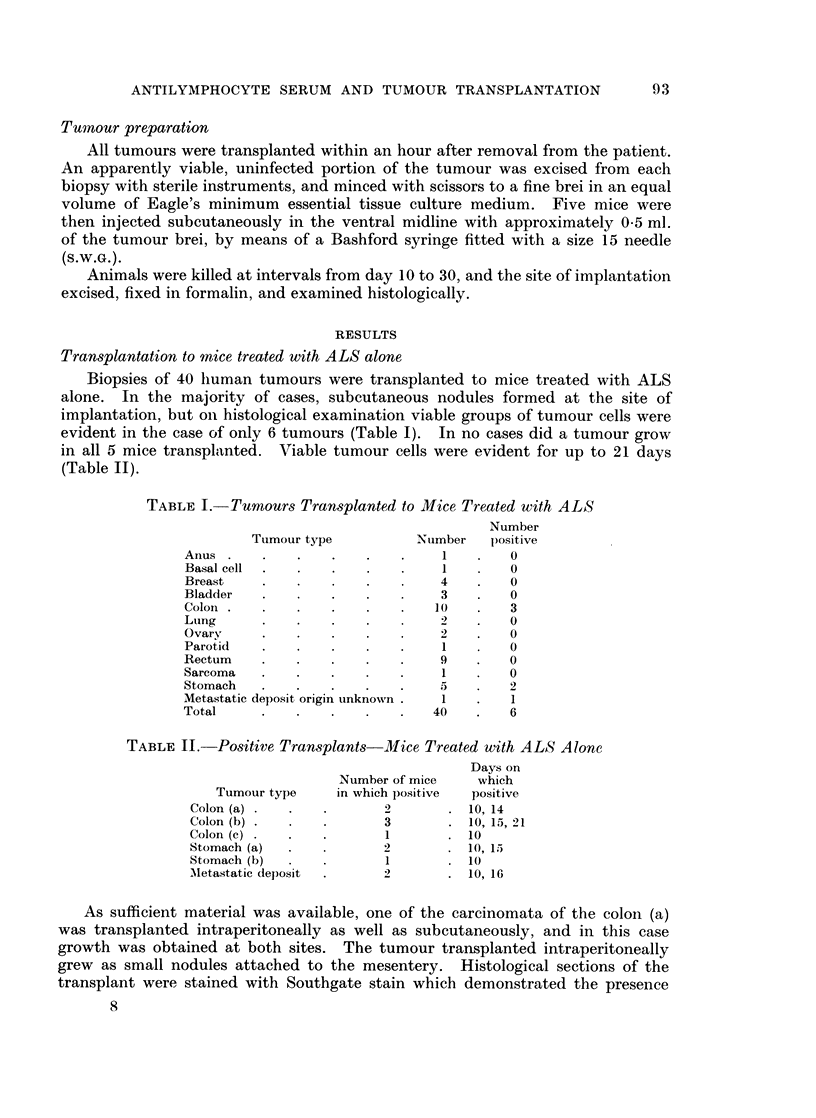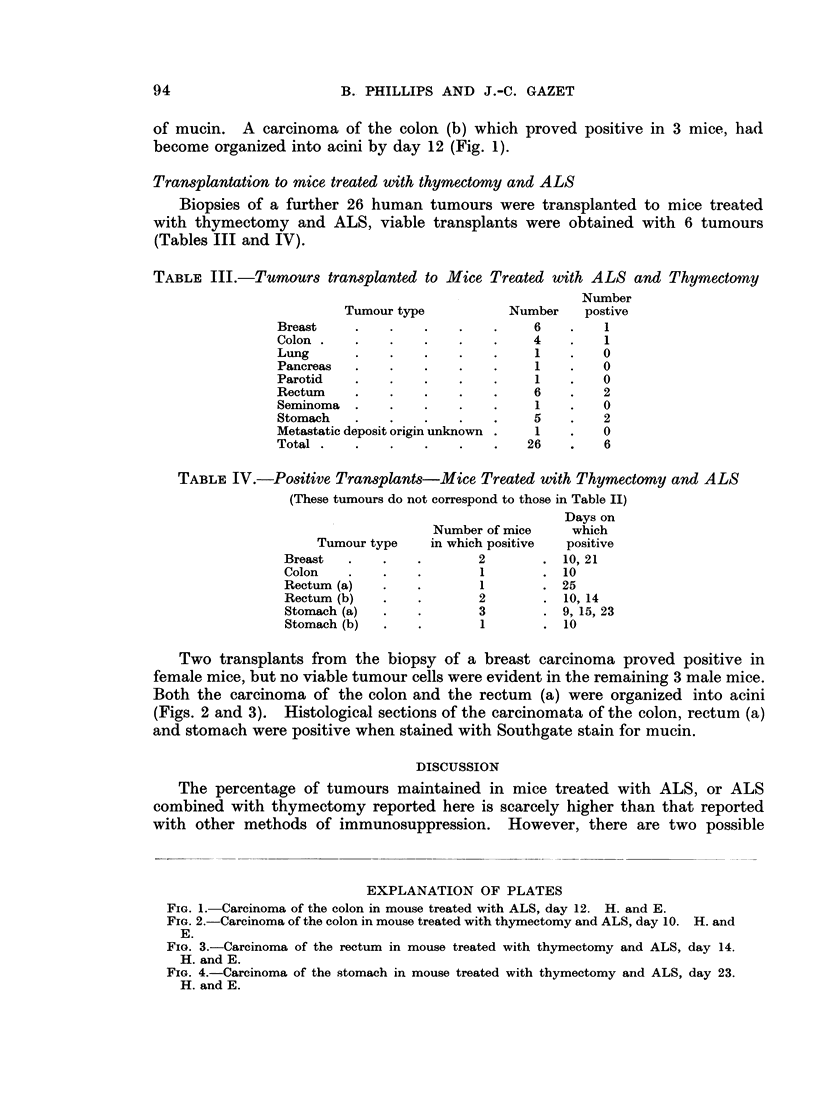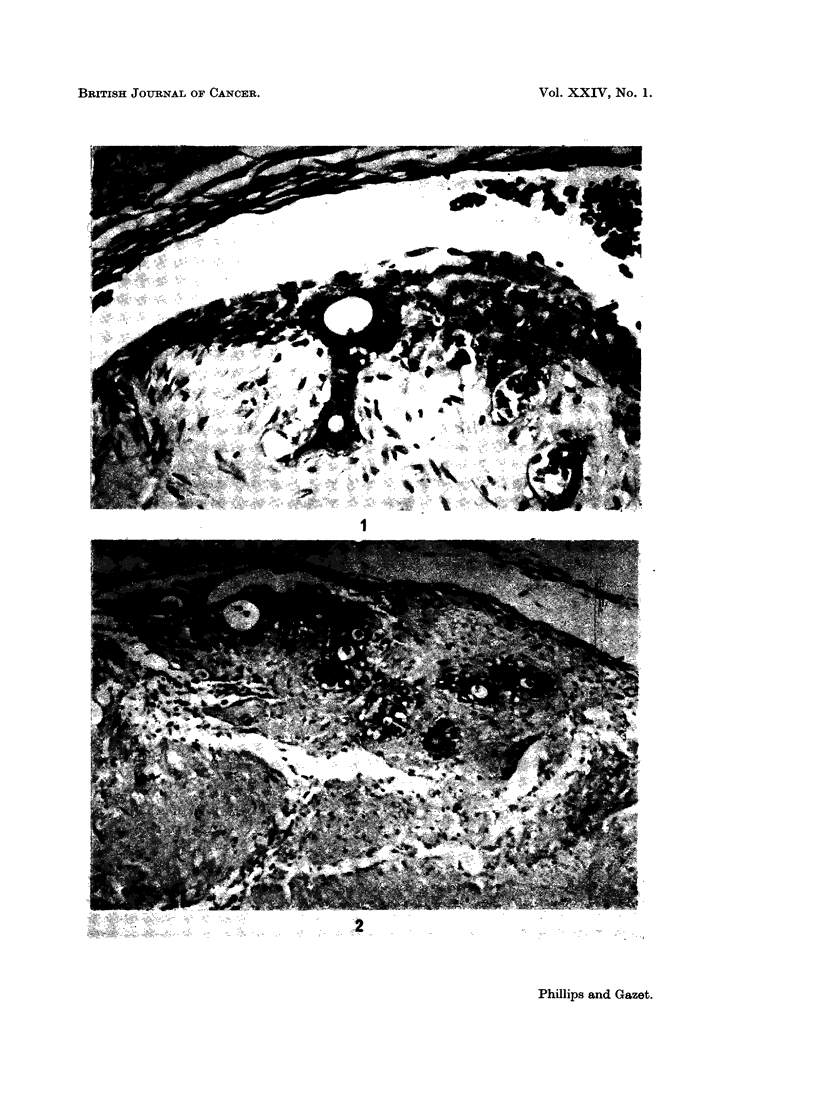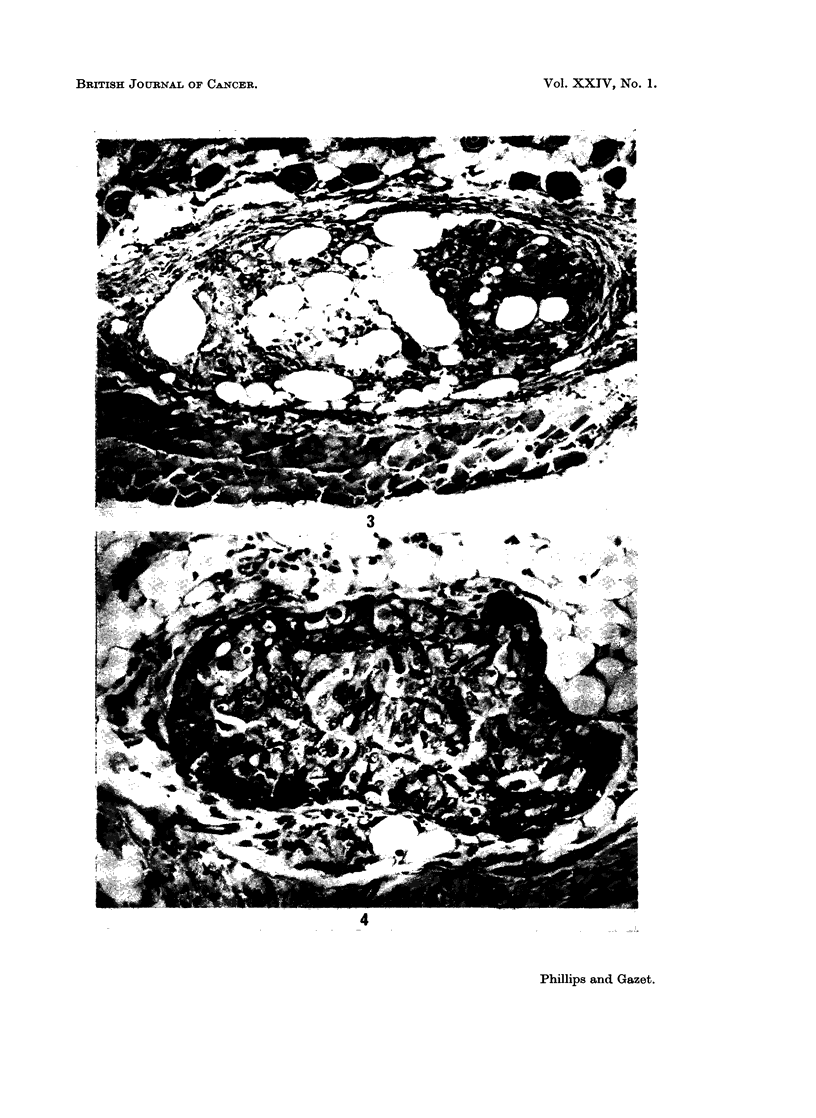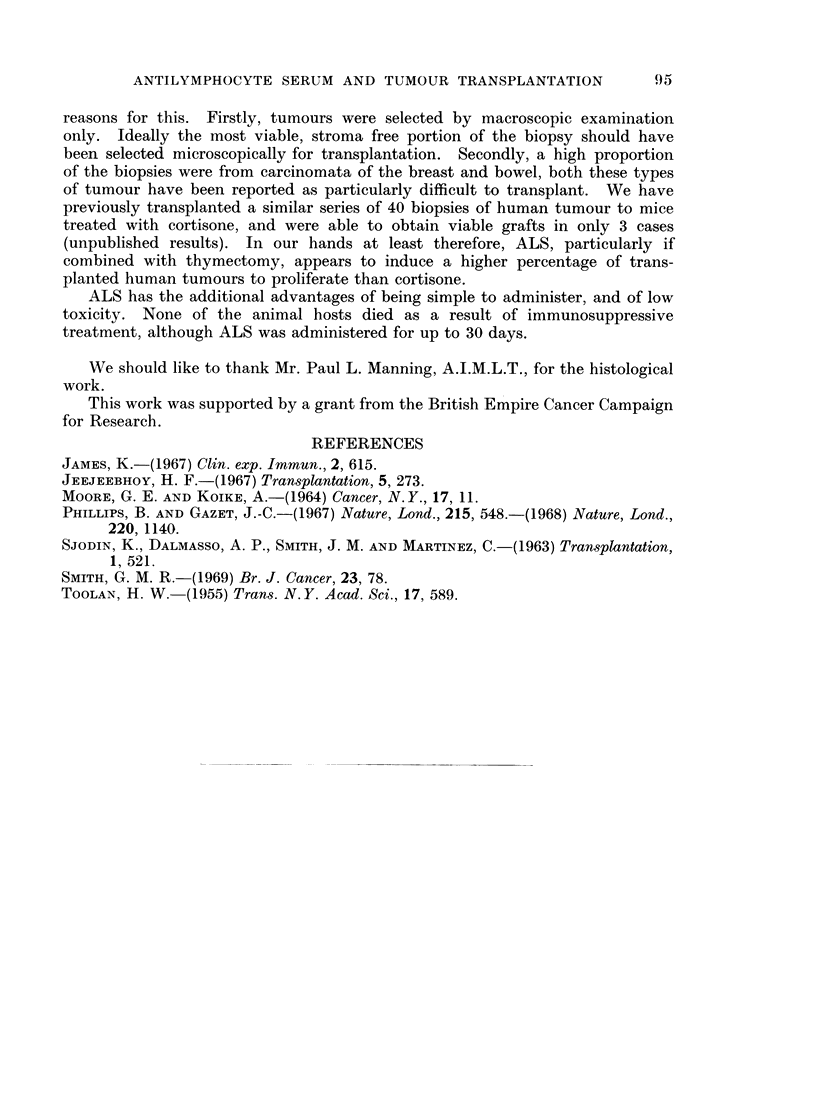# Transplantation of Primary Explants of Human Tumour to Mice Treated with Antilymphocyte Serum

**DOI:** 10.1038/bjc.1970.12

**Published:** 1970-03

**Authors:** B. Phillips, J. C. Gazet

## Abstract

**Images:**


### TRANSPLANTATION OF PRIMARY EXPLANTS OF HUMAN TUMOUR TO MICE TREATED WITH ANTILYMPHOCYTE SERUM

B. PHILLIPS AND J.-C. GAZET

*From the Surgical Unit, St. George's Hospital, Hyde Park Corner, London S.W.1*

Received for publication October 27, 1969

**SUMMARY.—Biopsies from 40 human tumours, obtained at operation, have been transplanted to mice treated with antilymphocyte serum (ALS); viable grafts were obtained from 6 tumours. A further 26 human tumours were transplanted to mice thymectomized as adults and treated with ALS; viable grafts were obtained in 6 cases.**

THERE are numerous reports of *in vivo* studies on human tumours transplanted to laboratory animals, in which the immune response has been partially suppressed by cortisone and irradiation (Toolan, 1955; Moore and Koike, 1964; Smith, 1969). However, only a small percentage of biopsies of human tumours obtained at operation can be maintained in this way, and even fewer can be induced to proliferate. An immunosuppressant which would induce a higher percentage of transplanted human tumours to proliferate would therefore be an advantage.

Antilymphocyte serum (ALS) has been shown to be an effective immunosuppressant (James, 1967), and we have previously demonstrated that human tumour cell lines HeLa, Hep2 and Hs1 will proliferate readily when transplanted subcutaneously to mice treated with ALS (Phillips and Gazet, 1968). It was consequently felt that ALS might prove of value in the transplantation of human tumour biopsies.

### METHODS

### *Animals*

Newly weaned Swiss mice of either sex were used. The mice were maintained as a closed colony.

### *Immunosuppression*

ALS was prepared in rabbits as previously described (Phillips and Gazet, 1967). Individual sera were tested by their ability to sustain the growth of the human tumour cell line HeLa in Swiss mice. Sera of adequate potency were pooled and stored at —20° C. The mice were injected subcutaneously in the nape of the neck with 0·5 ml. of ALS at the time of transplantation, and with 0·25 ml. of ALS on alternate days thereafter.

Thymectomy appears to enhance the action of ALS (Jeejeebhoy, 1967; Phillips and Gazet, 1968), therefore in some cases tumours were transplanted to thymectomized mice treated with ALS. The mice were thymectomized using the method described by Sjodin *et al.* (1963). Thymectomy was performed on 14-day-old animals since in our hands thymectomy at this age resulted in fewer mortalities due to technique than thymectomy of fully mature animals.

*Tumour preparation*

All tumours were transplanted within an hour after removal from the patient. An apparently viable, uninfected portion of the tumour was excised from each biopsy with sterile instruments, and minced with scissors to a fine brei in an equal volume of Eagle's minimum essential tissue culture medium. Five mice were then injected subcutaneously in the ventral midline with approximately 0·5 ml. of the tumour brei, by means of a Bashford syringe fitted with a size 15 needle (S.W.G.).

Animals were killed at intervals from day 10 to 30, and the site of implantation excised, fixed in formalin, and examined histologically.

### RESULTS

*Transplantation to mice treated with ALS alone*

Biopsies of 40 human tumours were transplanted to mice treated with ALS alone. In the majority of cases, subcutaneous nodules formed at the site of implantation, but on histological examination viable groups of tumour cells were evident in the case of only 6 tumours (Table I). In no cases did a tumour grow in all 5 mice transplanted. Viable tumour cells were evident for up to 21 days (Table II).

TABLE I.—*Tumours Transplanted to Mice Treated with ALS*

| Tumour type | Number | Number positive |
|---|---|---|
| Anus | 1 | 0 |
| Basal cell | 1 | 0 |
| Breast | 4 | 0 |
| Bladder | 3 | 0 |
| Colon | 10 | 3 |
| Lung | 2 | 0 |
| Ovary | 2 | 0 |
| Parotid | 1 | 0 |
| Rectum | 9 | 0 |
| Sarcoma | 1 | 0 |
| Stomach | 5 | 2 |
| Metastatic deposit origin unknown | 1 | 1 |
| Total | 40 | 6 |

TABLE II.—*Positive Transplants—Mice Treated with ALS Alone*

| Tumour type | Number of mice in which positive | Days on which positive |
|---|---|---|
| Colon (a) | 2 | 10, 14 |
| Colon (b) | 3 | 10, 15, 21 |
| Colon (c) | 1 | 10 |
| Stomach (a) | 2 | 10, 15 |
| Stomach (b) | 1 | 10 |
| Metastatic deposit | 2 | 10, 16 |

As sufficient material was available, one of the carcinomata of the colon (a) was transplanted intraperitoneally as well as subcutaneously, and in this case growth was obtained at both sites. The tumour transplanted intraperitoneally grew as small nodules attached to the mesentery. Histological sections of the transplant were stained with Southgate stain which demonstrated the presence of mucin. A carcinoma of the colon (b) which proved positive in 3 mice, had become organized into acini by day 12 (Fig. 1).

*Transplantation to mice treated with thymectomy and ALS*

Biopsies of a further 26 human tumours were transplanted to mice treated with thymectomy and ALS, viable transplants were obtained with 6 tumours (Tables III and IV).

TABLE III.—*Tumours transplanted to Mice Treated with ALS and Thymectomy*

| Tumour type | Number | Number postive |
|---|---|---|
| Breast | 6 | 1 |
| Colon | 4 | 1 |
| Lung | 1 | 0 |
| Pancreas | 1 | 0 |
| Parotid | 1 | 0 |
| Rectum | 6 | 2 |
| Seminoma | 1 | 0 |
| Stomach | 5 | 2 |
| Metastatic deposit origin unknown | 1 | 0 |
| Total | 26 | 6 |

TABLE IV.—*Positive Transplants—Mice Treated with Thymectomy and ALS*

(These tumours do not correspond to those in Table II)

| Tumour type | Number of mice in which positive | Days on which positive |
|---|---|---|
| Breast | 2 | 10, 21 |
| Colon | 1 | 10 |
| Rectum (a) | 1 | 25 |
| Rectum (b) | 2 | 10, 14 |
| Stomach (a) | 3 | 9, 15, 23 |
| Stomach (b) | 1 | 10 |

Two transplants from the biopsy of a breast carcinoma proved positive in female mice, but no viable tumour cells were evident in the remaining 3 male mice. Both the carcinoma of the colon and the rectum (a) were organized into acini (Figs. 2 and 3). Histological sections of the carcinomata of the colon, rectum (a) and stomach were positive when stained with Southgate stain for mucin.

### DISCUSSION

The percentage of tumours maintained in mice treated with ALS, or ALS combined with thymectomy reported here is scarcely higher than that reported with other methods of immunosuppression. However, there are two possible

---

EXPLANATION OF PLATES

FIG. 1.—Carcinoma of the colon in mouse treated with ALS, day 12. H. and E.

FIG. 2.—Carcinoma of the colon in mouse treated with thymectomy and ALS, day 10. H. and E.

FIG. 3.—Carcinoma of the rectum in mouse treated with thymectomy and ALS, day 14. H. and E.

FIG. 4.—Carcinoma of the stomach in mouse treated with thymectomy and ALS, day 23. H. and E.

1

2

Phillips and Gazet.

3

4

Phillips and Gazet.

reasons for this. Firstly, tumours were selected by macroscopic examination only. Ideally the most viable, stroma free portion of the biopsy should have been selected microscopically for transplantation. Secondly, a high proportion of the biopsies were from carcinomata of the breast and bowel, both these types of tumour have been reported as particularly difficult to transplant. We have previously transplanted a similar series of 40 biopsies of human tumour to mice treated with cortisone, and were able to obtain viable grafts in only 3 cases (unpublished results). In our hands at least therefore, ALS, particularly if combined with thymectomy, appears to induce a higher percentage of transplanted human tumours to proliferate than cortisone.

ALS has the additional advantages of being simple to administer, and of low toxicity. None of the animal hosts died as a result of immunosuppressive treatment, although ALS was administered for up to 30 days.

We should like to thank Mr. Paul L. Manning, A.I.M.L.T., for the histological work.

This work was supported by a grant from the British Empire Cancer Campaign for Research.

### REFERENCES

James, K.—(1967) *Clin. exp. Immun.*, **2**, 615.

Jeejeebhoy, H. F.—(1967) *Transplantation*, **5**, 273.

Moore, G. E. and Koike, A.—(1964) *Cancer, N.Y.*, **17**, 11.

Phillips, B. and Gazet, J.-C.—(1967) *Nature, Lond.*, **215**, 548.—(1968) *Nature, Lond.*, **220**, 1140.

Sjodin, K., Dalmasso, A. P., Smith, J. M. and Martinez, C.—(1963) *Transplantation*, **1**, 521.

Smith, G. M. R.—(1969) *Br. J. Cancer*, **23**, 78.

Toolan, H. W.—(1955) *Trans. N.Y. Acad. Sci.*, **17**, 589.